# Sex differences in uric acid and NT-pro BNP assessments during coronary severity

**DOI:** 10.1097/MD.0000000000019653

**Published:** 2020-04-10

**Authors:** Guofeng Guo, Zhaoqi Huang, Shixiang Wang, Ximing Chen

**Affiliations:** Department of Cardiology, the third affiliated Hospital of Guangzhou Medical University, Guangdong Province, China.

**Keywords:** acute coronary syndrome, sex, NT-proBNP, severity of coronary artery disease, uric acid

## Abstract

To investigate the correlation between uric acid (UA) and N-terminal pro-brain natriuretic peptide (NT-proBNP) levels and coronary artery severity in acute coronary syndrome patients of different sexes.

A total of 134 patients with acute coronary syndrome (ACS) were investigated. According to sex, there were 96 cases in male group and 38 cases in female group. According to the number of diseased vessels, the degree of coronary artery lesion was determined and divided into negative group (n = 21), single vessel lesion group (n = 43), double vessel lesion group (n = 38), and 3 vessel lesion group (n = 32).

Univariate analysis showed that UA, NT-proBNP was correlated with the severity of ACS (*P* < .05). UA was an independent risk factor for the severity of coronary artery disease in female group (*P* < .05), but not in male group (*P* > .05). There was no significant correlation between NT-proBNP and severity of coronary artery disease in different sex (*P* > .05).

UA was significantly correlated with the severity of coronary heart disease, especially in women, but not in men. The level of NT-proBNP was positively correlated with the severity of coronary artery, but no significant difference was found in different sexes.

## 1 Introduction

Acute coronary syndrome (ACS) presents a high incidence and mortality,^[1]^ which is the main cause of death worldwide.^[2]^ Every year, about 2.5 million patients with chest pain worldwide are diagnosed with ACS.^[3]^ A number of studies have shown that uric acid (UA) is closely related to CAD, especially to the severity of coronary artery disease, while some studies suggest that the correlation exists only in women.^4^,^5^ Many studies have proved that the severity of coronary artery in patients with ACS is positively correlated with the level of N-terminal pro-brain natriuretic peptide (NT-proBNP), especially in the study of BIOMARCS.^6^,^7^,^8^ The level of NT-proBNP in women is higher than that in men may be related to sex hormones,^9^,^10^ and sex hormones are the protective factor of coronary artery. There is no study on the correlation between NT-proBNP level and coronary artery severity in different sexes. Therefore, UA and NT-proBNP are similar in sex and coronary artery severity, so this study is to explore the correlation between UA and NT-proBNP levels and coronary artery severity in ACS patients of different genders.

## 2 Materials and methods

### 2.1 Study design and patient population

From May 2018 to March 2019, 134 patients with ACS diagnosed by coronary angiography were analyzed retrospectively in the third affiliated Hospital of Guangzhou Medical University.

### 2.2 Methodology

Experienced clinicians systematically enquire and record demographics and disease history for all individuals. Diabetes was considered present if there was a documented diagnosis requiring treatment with medication or diet, and hypertension was considered present if there was a documented history of hypertension treated with medication. Creatine kinase isoenzyme (CK-MB) and troponin (cTnI) were measured immediately after admission. The next day, venous blood was taken from the patients who were on an empty stomach for blood biochemical tests. Such as triglyceride (TG), total cholesterol (TC), high-density lipoprotein cholesterol (HDL-C), low-density lipoprotein cholesterol (LDL-C), UA, and NT-proBNP were detected and left ventricular ejection fraction(LVEF) was determined by echocardiography. According to cag results, assessed by at least 2 experienced cardiovascular specialists, the degree of left and right coronary artery lesions <50% was defined as negative. According to the number of coronary artery lesions, the lesions were divided into single vessel, double vessel, and 3 vessel lesions.

### 2.3 Statistical analysis

Continuous data are expressed as mean ± standard deviation or median (interquartile range), and categorical data as percentages. Analysis of variance (ANOVA) and nonparametric statistics such as the chi-squared test were used for continuous and categorical variables, respectively. Linear-by-Linear association was used for linear trend test. Multivariate logistic regression analysis was used to assess the effects of UA, NT-proBNP, LDL-C, LVEF, and cTnI. Statistical analysis was performed using the Statistical Package for Social Sciences (SPSS) for Windows (version 25; SPSS Inc., Chicago, IL), and a 2-tailed *P* < .05 was considered statistically significant.

## 3 Results

The baseline clinical characteristics of the study population are shown in Table 1.

**Table 1:**
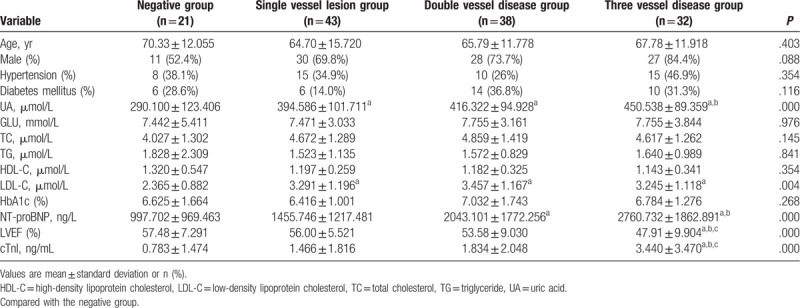
Baseline characteristics.

A total of 134 patients were enrolled, including 96 cases in male group (mean age 63.58 ± 12.728 years) and 38 cases in female group (mean age 74.32 ± 11.404 years). According to the results of coronary angiography, there were 48 cases in negative group, 22 cases in single vessel lesion group, 46 cases in double vessel lesion group, and 32 cases in 3 vessel lesion group.

There was no significant difference in age, sex, history of hypertension, history of diabetes, blood glucose, total cholesterol, TC, TG, HDL-C, and HbA1c among the 4 groups (*P* > .05). Compared with the negative group, the difference in UA and LDL-C was statistically significant in the single lesion group (^a^ *P* < .05); the difference in UA, LDL-C, and NT-proBNP in the double lesion group (^a^ *P* < .05); and the 3 lesion groups were statistically significant in UA, LDL-C, NT-proBNP, LVEF, and cTnI (^a^ *P* < .05). Compared with the single-branch lesion group, the 3 lesion groups were statistically significant in terms of UA, NT-proBNP, LVEF, and cTnI (^b^ *P* < .05). Compared with the 2-branch lesion group, the 3 lesion groups were statistically significant in LVEF and cTnI (^c^ *P* < .05).

Table 2 shows the men and women were divided into groups, and the correlation between UA, LDL-C, NT-proBNP, LVEF, and cTnI and the severity of coronary artery disease was counted in different sex groups.

**Table 2:**
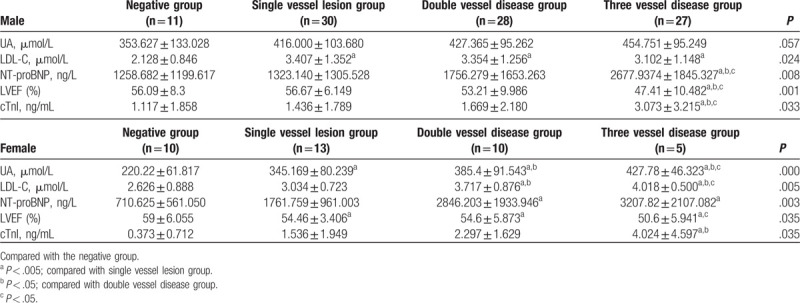
Comparison of relevant data between male and female groups.

There was no significant difference in UA in male group (*P* > .05). In LDL-C, compared with the negative group, there were statistically significant differences in single lesion group, double lesion group, and 3 lesion group (^a^ *P* < .05). Compared with the negative group, the single branch lesion group, and the double branch lesion group, the 3 lesion groups were statistically significant in NT-proBNP, LVEF, and cTnI (^a^ *P* < .05, ^b^ *P* < .05, ^c^ *P* < .05).

In the female group, there was significant difference in UA between the 4 groups (^a^ *P* < .05). In LDL-C, compared with the negative group and the single vessel lesion group, there was significant difference between the 2 groups and the 3 vessel disease group (^a^ *P* < .05, ^b^ *P* < .05). Compared with the double branch disease group, the difference of the 3 branch disease group was also statistically significant (^c^ *P* < .05). In terms of NT-proBNP, compared with the negative group, there was significant difference between the 2-vessel lesion group and the 3-vessel lesion group (^a^ *P* < .05). In LVEF, compared with the negative group, the single-vessel lesion group, there was significant difference between the 2-vessel lesion group and the 3-vessel lesion group (^a^ *P* < .05). There was also a statistical difference between the 3 lesions compared with the 2 lesions group (^c^ *P* < .05). In cTnI, the 3 lesions were statistically significant compared with the negative group and the single lesion group (^a^ *P* < .05, ^b^ *P* < .05).

Both male and female groups were treated with ordered multi-classification Logistic regression method, and the extent of coronary artery lesion was taken as dependent variable. Univariate analysis showed that UA, NT-proBNP, LDL-C, LVEF, cTnI, and other indexes were correlated with the severity of ACS (*P* < .05). These related indexes were taken as independent variables for analysis. Table 3 shows that LVEF and cTnI were independently correlated with the severity of coronary lesions in different sexes (*P* < .05); UA and LDL-C were independently correlated with the severity of coronary artery disease in the female group (*P* < .05), but not in the male group (*P* > .05). There was no significant correlation between NT-proBNP and the severity of coronary artery disease in different sexes (*P* > .05).

**Table 3:**
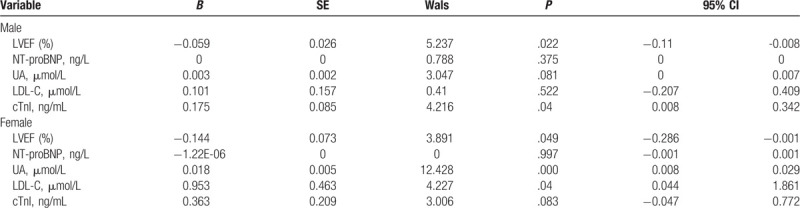
Logistic regression analysis of the degree of coronary artery disease and other factors in male and female.

## 4 Discussion

UA is the final metabolite of purine nucleotides,^[11]^ which is formed by the decomposition of adenosine and guanine. Purine comes from both endogenesis (purine synthesis and nucleic acid decomposition) and exogenous (absorption from the outside world). Purine degradation can lead to the formation of free radicals, platelet adhesion, and aggregation. High levels of UA are also associated with endothelial dysfunction, smooth muscle proliferation, and changes in nitric oxide (NO) production, and are also considered to be markers of oxidative stress and inflammation.^[12]^ In the early stages of atherosclerosis, UA is an antioxidant that occurs in the process of atherosclerosis when hyperuricemia occurs. It will be reversed to oxidative state and directly involved in the process of inflammation and atherosclerosis.

The relationship between UA level and cardiovascular disease has been discussed in recent years. Many studies at home and abroad have confirmed that hyperuricemia is significantly correlated with CAD^13^,^14^,^15^,^16^ and positively correlated with coronary artery severity.^4^,^13^,^15^ Other studies have shown that the level of UA is closely related to age and age-related changes. It varies with age and sex, but the mechanism of UA level with age and sex is not clear.^[14]^ There is an independent association between hyperuricemia and coronary heart disease in women, and there is a trend that the higher the level of UA, the 3 vessels. The higher the incidence of change, the higher the incidence of the analysis of male and female data, the study found that this trend appears only in women, consistent with the results of this study. But for coronary heart disease, whether UA is an independent risk factor is still controversial. UA is closely related to the risk factors of coronary heart disease, such as hypertension, diabetes, and dyslipidemia. These studies suggest that the association between the UA and the CAD may be related to the link between the UA and the risk factors of the CAD. A meta-analysis shows that hyperuricemia may increase the risk of coronary heart disease, regardless of the traditional CAD risk factors.^[17]^ In this study, because of the small sample size and many confounding factors, other related factors can not be excluded from the results of this study.

BNP^[10]^ is a kind of neurohormone secreted by ventricular myocytes after myocardial cell ischemia, injury, necrosis, and increase of ventricular wall tension, which has diuretic sodium excretion, vasodilation, anti-cardiac remodeling, and renin-angiotensin system system. It was originally isolated from pig brain tissue by Japanese scholars and is called brain natriuretic peptide. BNP and NT-proBNP are internationally recognized biomarkers of cardiac function.^[18]^ However, the biological half-life of BNP in peripheral blood was shorter than that of NT-proBNP, in peripheral blood, so the concentration of BNP in peripheral blood was also lower than that in NT-proBNP, so NT-proBNP was more widely used.^[7]^

At present, the study on the severity of NT-proBNP and ACS is increasing. Many studies^6^,^7^,^8^ have found that the level of NT-proBNP is positively correlated with the level of cTnI and increases with the severity of the coronary artery disease. The study also showed that the higher the number of coronary artery lesions, the higher the plasma NT-proBNP level and the NT-proBNP level in the anterior descending coronary artery disease group was significantly higher than that in the right coronary artery and circumflex artery disease group. This study confirmed that the plasma NT-proBNP level can be used to judge the severity of myocardial ischemia. NT-proBNP levels in women are higher than those in men, which may be related to sex hormones,^9^,^10^ which are protective factors of coronary artery.^[19]^ However, some studies^[20]^ suggest that there is no significant difference in NT-proBNP levels between men and women, which may be due to the stimulating effect of female sex hormones on natriuretic peptide gene expression. UA and NT-proBNP have common characteristics in terms of sex and coronary heart disease. However, currently there is no study on the correlation between NT-proBNP level and coronary artery severity in ACS patients of different sexes. In this study, ACS patients were divided into male and female groups according to the degree of coronary artery disease. Univariate and Logistic analysis showed that NT-proBNP level was positively correlated with coronary artery severity, but no significant difference was found in different sexes.

## 5 Conclusion

Hyperuricemia was significantly correlated with the severity of coronary artery disease only in women, while the level of NT-proBNP increased with the severity of coronary artery disease regardless of sex.

## Author contributions

**Conceptualization:** Zhaoqi Huang.

**Data curation:** Guofeng Guo.

**Formal analysis:** Shixiang Wang.

**Methodology:** Zhaoqi Huang.

**Project administration:** Ximing Chen.

**Resources:** Guofeng Guo.

**Software:** Guofeng Guo.

**Validation:** Ximing Chen.

**Writing – original draft:** Guofeng Guo.

Guofeng Guo orcid: 0000-0003-3153-0306.
